# The Upcoming Role for Nursing and Assistive Robotics: Opportunities and Challenges Ahead

**DOI:** 10.3389/fdgth.2020.585656

**Published:** 2020-12-01

**Authors:** Eftychios G. Christoforou, Sotiris Avgousti, Nacim Ramdani, Cyril Novales, Andreas S. Panayides

**Affiliations:** ^1^Department of Mechanical and Manufacturing Engineering, University of Cyprus, Nicosia, Cyprus; ^2^Department of Nursing, School of Health Sciences, Cyprus University of Technology, Limassol, Cyprus; ^3^Université d'Orléans, INSA CVL, PRISME EA 4229, Orléans, France; ^4^Department of Computer Science, University of Cyprus, Nicosia, Cyprus

**Keywords:** nursing robots, socially-assistive robots, physically-assistive robots, healthcare robotics, connected health, robotics, eHealth

## Abstract

As an integral part of patient care, nursing is required to constantly adapt to changes in the healthcare system, as well as the wider financial and societal environment. Among the key factors driving these changes is the aging of population. Combined with an existing shortage of nursing and caregiving professionals, accommodating for the patients and elderly needs within hospitals, elderly-care facilities and at a home setting, becomes a societal challenge. Amongst the technological solutions that have evolved in response to these developments, nursing and assistive robotics claim a pivotal role. The objective of the present study is to provide an overview of today's landscape in nursing and assistive robotics, highlighting the benefits associated with adopting such solutions in standard clinical practice. At the same time, to identify existing challenges and limitations that essentially outline the area's future directions. Beyond technological innovation, the manuscript also investigates the end-users' angle, being a crucial parameter in the success of robotics solutions operating within a healthcare environment. In this direction, the results of a survey designed to capture the nursing professionals' perspective toward more informed robotics design and development are presented.

## Introduction

Nurses constitute the backbone of the healthcare industry and the nursing profession itself has typically been the largest segment of the healthcare workforce. Steadily rising healthcare costs and a population that is gradually aging are both factors impacting the healthcare systems and the nursing profession. A notable fact is that population aging is becoming a global phenomenon with wider financial and social implications. In the European Union alone, it is projected that older people (≥65) will rise from 101 million in 2018 to 149 million by 2050. From a percentage angle, there will be an increase of 17.6 and 60.5% of people aged between 65–74 and 75–84, respectively in the EU-28, while the highest expansion growth is expected for very old people (≥85) at a rate of 130.3%. On the opposite end, people aged <55 years will shrink by 9.6% during this period. At the same time, the old age dependency ratio (OADR) is expected to climb from 30.5% in 2018 to 49.9% in 2050 (i.e., from ~three persons of working age between 15 and 64 for every older adult in 2018 to two persons in 2050), with the global OADR projected to reach 28% (1, 2). At a personal level, elderly individuals are challenged in various aspects including social (neglect, isolation, fear, loneliness, boredom), financial (low income, fear of becoming a burden, lack of insurance), psychological (depression, poor memory, dementia, insomnia), physiological (decline of mental abilities, less efficient reflexes, muscle weakness, weak body balance, falls, fragile bones) (3). For the above reasons, older people require special care that friends and relatives are often unable to provide and this commonly leads to institutionalization.

In response to the existing shortage of nursing and caregiving professionals, along with the rising healthcare costs, the employment of various technological solutions has been proposed. Technologies which have evolved to support the independent living and aging-in-place concepts include “Ambient Assisted Living” (4). The purpose of these technologies, also referred to as “smart home” technologies, is to support independent living using a combination of sensors appropriately installed in a house setup (stationary or wearable). Such sensors include magnetic switches, temperature sensors, photosensors, water flow sensors, motion sensors, force sensors, smoke detectors, and biosensors for vital signs (5). Ambient monitoring systems may capture activities of everyday living, which can then be exploited in two distinct ways: identify short-term emergencies; identify long-term variations in health status (6). Despite the relevance of these technologies to patient and elderly care, further consideration is beyond the scope of the present work, which focuses on robotics-related technologies.

From a robotics perspective, specially-designed systems have the potential to ease the burden on nursing staff within hospitals and nursing homes but also to undertake general assistive roles at home, without compromising quality of care while improving quality of life. Consistent to the above roles is the distinction between nursing and assistive robots:

- *Nursing robots* may serve as supplemental healthcare workers in hospitals, elderly-care facilities, and at home. They can perform logistics and laborious physical tasks, combat loneliness and inactivity in the elderly population, or assigned routine tasks such as measuring patients' vital signs. Remote-controlled telerobots can handle interactive caretaker duties and serve as interfaces for doctors and/or nurses to communicate with patients and/or the elderly over distance.
- *Assistive robots* may enable disabled and/or elderly people to pursue healthy, independent and productive lives. Depending on their primary role, assistive robots are grouped into: “Socially-assistive” and “Physically-assistive.” The former, provide assistance to end-users through social interaction while the latter through physical interaction.

Enhanced capabilities for the above robotics technologies exist within the wider scope of telerobotics and telemedicine. Nursing and assistive robots are in fact part of the wider field of healthcare robotics, which also include the medical robotic systems. The latter has been an area of active research and various systems have already been established in clinical practice. Robotic systems are currently involved in surgical specialties including general surgery, orthopedic and neurosurgery, as well as other therapeutic procedures, such as radiation treatments (7). Realistically, employment of nursing and assistive robotics involves numerous challenges: technological, clinical, financial, insurance, psychological, social, ethical and legal. From a technological perspective challenges include indoor navigation, manipulation, safety, telecommunications, and integration of robots with existing in-hospital technologies. Key integration examples involve the connection to the hospitals' enterprise resource planning (ERP) and electronic health records (EHR) software systems. Corresponding tasks for nursing robots include performing logistics operations and vital signs measurements, respectively. On the other end, user perceptions and attitudes toward nursing and assistive robots are expected to have a decisive role on the future and the impact of these technologies. This is relevant both from the patients and the elderly point-of-view, as well as the nursing professionals and caregivers (8).

The purpose of this work is to provide an overview of the emerging fields of nursing and assistive robotics in order to highlight their potential and identify the involved challenges. The latter provides guidelines for robot design and directions for future developments in these areas. Informed design may constitute robotic solutions more usable and effective allowing them to better serve their purpose. The paper is organized as follows. It starts with an overview of nursing robots in section Nursing Robots and the discussion extends to socially-assistive and physically-assistive robots in sections Socially-Assistive Robots and Physically-Assistive Robots, respectively. In these sections, the added-value and potential of these robotic solutions are portrayed. Then, the enhancements in nursing and assistive robots facilitated via the integration with telerobotics technologies is highlighted in section A Role for Telerobotics. The emerging robotics role in disease outbreaks is discussed in section Robots in Times of Disease Outbreaks. Section Robots in Healthcare Environments: Endorse Concept Case Study examines the introduction of robots in the healthcare environment through a case study of the EU-funded ENDORSE project and a survey designed to capture end-user views upon different aspects of nursing robots. Challenges pertinent to future developments in the areas of nursing and assistive robotics are the topic of section The Challenges Ahead. The last section presents the conclusions.

## Nursing Robots

A role for nursing robots exists both in hospitals and elderly-care facilities. Robots may effectively relieve burden from nurses allowing them to concentrate on tasks pertinent to their primary duties. Robotic machines have already been considered to support processes including distribution of food trays, medicines, and laboratory specimens throughout a hospital. Robots may also automate logistics tasks relevant to medical equipment and supply storage. Beyond these tasks, an upgraded role for robots includes working alongside or collaborating with nurses to support their work and enhance efficiency. Moreover, robot nurses can help reduce occupational exposure of human nurses to hazardous infections or chemicals. Following special training, nurses may undertake the role of coordinating and overseeing the duties of a robotic fleet within a hospital; thus, creating a new professional specialization.

Specially-designed robotic systems that help with patient transfers, ambulation, and lifting may significantly reduce physical stress on nurses. It is common for caregivers to suffer from back pain and job-related illnesses. Specially-designed robotic devices may be assigned laborious tasks, such as transferring and moving patients (9). This aspect also directs to the wider research on wearable exoskeleton devices. Exoskeletons may enhance a person's physical capabilities allowing lifting of heavier weights (power extenders), while preventing musculoskeletal disorders. In fact, exoskeletons provide an alternative to fully-automated robotic solutions, effectively preserving the human skills in the job.

Nursing robots may also provide services for telemedicine purposes (10). Robotic nurses accommodating telepresence platforms can effectively serve as interfaces for doctors to communicate with patients over distance. Typical scenarios involve routine virtual visits where the robot navigates to hospital wards employing the onboard screen to establish the required visual contact with the examined patients. Toward this direction, endowing robots with autonomous navigation capabilities is a particularly attractive feature, which relieves the necessity of operators manually navigating robots until a specific patient is located. Additionally, the robot may also capture the patient's vital signs at various intervals as required for a diagnosis and typical clinical protocols. In principle, the latter scenario further extends to the patient's home setup bringing specialized care to citizens and healthcare centers situated in remote and isolated areas.

Overall, electromechanical caregivers have unique advantages over their human counterparts including the capacity to work continuously throughout the day. Being programmable machines, robots have the potential to personalize care and adapt to varying needs. Importantly, robots can be integrated with other hospital technologies, such as cloud-based EHR systems, facilitating access to a patient's complete medical history and thus ensuring continuity of care.

## Socially-Assistive Robots

A socially-assistive robot is a type of assistive robot, which provides assistance to end-users through social interaction (11). A natural human tendency to attribute human characteristics and intentions to mobile physical entities, constitutes robots more effective than any computer program or a smartphone mHealth application. Potential uses of socially-assistive robots suggested in literature are discussed below and include: (i) companion robots; (ii) supporting adults with dementia; (iii) motivating physical exercise; and (iv) providing post-stroke rehabilitation.

Companion robots have emerged as a special category within assistive robotics. A primary role has been to act as interfaces for the elderly to enrich their social lives, while connecting with their families and friends (12). Among the capabilities of socially-assistive robots is to monitor elderly patients via video and also provide alerts to caregivers on patient activity. Moreover, robots may provide older citizens with news and entertainment information, reminders for medication adherence, as well as facilitate physical exercise. On a different note, robotic pets have received considerable attention in an attempt to reduce stress and depression, while avoiding the effort and risks involved in animal care (11). Another key area of socially-assistive robots concerns supporting people suffering from dementia (13–16).

Regular physical exercise is essential in elderly individuals to maintain and improve health status, support mental and physical well-being, and reduce the likelihood of depression. Robots have been designed to engage elderly users in physical exercise (17) facilitating workout sessions, while evaluating user performance and providing real-time feedback. Two potential implementation challenges for these robots were identified in Görer et al. (18). First, is the automatic analysis of the coach's gestures toward being adequately reproduced, and second, is the different physical embodiment that a robot possesses compared to the coach. Use of robotics also extents to post-stroke exercising and rehabilitation, which typically involves carefully designed repetitive, passive or active exercises (11). In either case, a movement therapy robot may provide a diagnostic (measurement and assessment) or therapeutic (improvement of function) benefit.

## Physically-Assistive Robots

Two key elements of independent living that are directly associated to quality of life of both the elderly and patients (19) are: (a) The preservation of mobility; (b) The ability to manipulate objects. In elderly populations, a wide variety of medical conditions ranging from strokes and neurodegenerative diseases, to bone fractures and decline of muscular power, lead to the loss of mobility. To combat this situation, robotic solutions have been proposed to provide assistance required to stand-up, sit and walk (20). Robotic wheelchairs provide users with autonomy, enhanced mobility and safety (21). With an appropriate mechanical structure for the robotic wheelchair, architectural barriers may be overcome, including curb ascending and descending (22). In terms of control, a robotic wheelchair may hierarchically combine: (i) low-level functions (e.g., obstacle/collision avoidance, corridor centering) and (ii) high-level functions (e.g., directing the wheelchair) (23).

Appropriately-designed assistive robotic manipulation systems can support people with motor impairments such as limited hand and arm movements, high-level spinal injuries or tremors. Surveys have identified the relevant needs of disabled people in this group regarding assistive devices to carry out activities (23): eating and drinking (feeding assistive devices); personal care (washing, shaving, applying cosmetics); handling objects (books, devices); mobility and access (opening doors); general reaching and moving tasks. Manipulation systems addressing aforementioned challenges can be either fixed or wheelchair-mounted (24).

## A Role for Telerobotics

Within the wider field of healthcare, teleoperated medical robotic systems have been successfully employed allowing procedures such as surgeries, treatments, and diagnoses to be conducted across distances, while utilizing wired and/or wireless communication networks. Recent developments in telerobotics and their enabling technologies [robotic manipulation, video streaming; (25), telecommunications] constitute nursing and assistive robots more effective and widen their application fields (26). Robotics hardware enhances telepresence to a more natural and effective level through mobility and performance of manipulation tasks in the remote environment. Telerobotics solutions pertinent to nursing may facilitate the doctors' virtual visits scenario. Using an onboard adjustable camera, the user may remotely drive the robot to locate a patient in the clinic and/or provide a set of destination points to which the robot will autonomously navigate. Bidirectional video conferencing then allows the doctor to appear on the robot's screen and engage in a dialogue with the patient to assess his/her current clinical status (telehealth). Real-time medical charts can further complement and enhance this remote clinical assessment using a robot-mounted device equipped with vital signs acquisition capabilities and EHR connectivity, such as the ENDORSE concept discussed in section Robots in Healthcare Environments: Endorse Concept Case Study.

Telepresence robots supporting elderly persons at home may facilitate social interaction, help the elderly to remain socially engaged, and allow relatives to make virtual visits and experience the feeling of close proximity. They also enable contact with doctors and nurses to remotely monitor their health and provide the required support. Compared to video calls, a telepresence robot enhances interaction to a more natural level through the mobility of the system. Enhanced capabilities for telerobotic systems are possible through their inherent compatibility with IT technologies, including internet-of-things (IoT), as for example the IoT-enabled telerobotics application in home care proposed in Zhou et al. (27). In (28), various telepresence robotic systems are reviewed and three main areas of application of telerobotics in elderly care become apparent, namely: telemedicine, remote interactions with other people, and telehealth monitoring.

## Robots in Times of Disease Outbreaks

In times of outbreaks of contagious diseases, healthcare workers are in high risk for infection due to direct contact with patients. This exposure can be minimized when robots undertake some nursing duties (29). In that case, nursing robots play analogous roles to emergency response robots deployed in contaminated sites (e.g., following a nuclear plant accident). In either case, robots become frontline actors preventing human exposure to health hazards. Through the novel coronavirus crisis (COVID-19) in 2020 has emerged a renewed interest in robotics solutions as effective resources to combat a pandemic (30). Despite the research and development in the fields of nursing and service robotics, the robotics community was found unprepared to drastically deploy effective solutions following COVID-19 pandemic. However, an upgraded role for nursing robots has emerged regarding their potential to reduce personal physical contact and exposure. Various robot applications have been identified including autonomous robots deployed to disinfect hospital wards using non-contact ultraviolet (UV) surface disinfection methods, deliver medicine, food trays and medical supplies, or handling of contaminated waste within a hospital. An indirect benefit is that robots help reduce the usage (and need for reuse) of personal protective equipment and also avoid contamination during its removal.

When large-scale screening programs are implemented, robots may contribute in the collection of samples, while limiting physical contact and increasing the coverage of a study. Robotic manipulation systems can then be employed in laboratory testing by automating the processing of large sample quantities. For diagnosis and screening purposes robots can also undertake temperature measurements in public areas and ports of entry.

The previously mentioned roles envisioned for assistive robotics (see sections Nursing Robots, Socially-Assistive Robots, Physically-Assistive Robots, A Role for Telerobotics) also become relevant toward addressing quarantine and social distancing implications. Socially assistive robots may provide patients and elderly with companionship and sustain social contact, while physical visits are not possible. Also, robots may physically support elderly/patients at home and facilitate health monitoring when family, friends or caregivers become less available. Rehabilitation therapies may continue without the physical presence of a physiotherapist, and physical exercise sessions at home or elderly care facilities can be carried out without an instructor. On a different note, mobile robots can be used to supervise social distancing rules in public areas, check usage of protective equipment and provide reminders and alerts. Furthermore, ground or aerial robotic vehicles can assist in policing quarantine areas and border control operations.

Moreover, teleoperated robotic manipulation systems can play a role in diagnosis and health monitoring without physical presence of a medical expert. In particular, telesonography robots [e.g., (31, 32)] can be used for pulmonary condition examinations and prevent cross-contamination in suspected patients. Recently, a telerobotic ultrasound system was considered for cardiopulmonary assessment of COVID-19 patients, as presented in Zhou et al. (27).

## Robots in Healthcare Environments: Endorse Concept Case Study

Despite the documented clinical value and commercial potential, there is a limited market penetration of mobile robotic solutions in hospital environments today. The latter is further amplified by the fact that only a handful of solutions exist, which are purpose-oriented and do not adequately scale to accommodate the wide range and high demand of clinical services and logistics tasks that would translate into wider adoption. Moreover, vendors often overlook the importance and necessity of integrating their solutions with existing healthcare systems, while robotic fleets are vulnerable to cybersecurity attacks, and typically involve time consuming and costly infrastructure setups (33–35). These areas are currently attracting considerable research interest worldwide.

### The ENDORSE Concept

**Figure 1:**
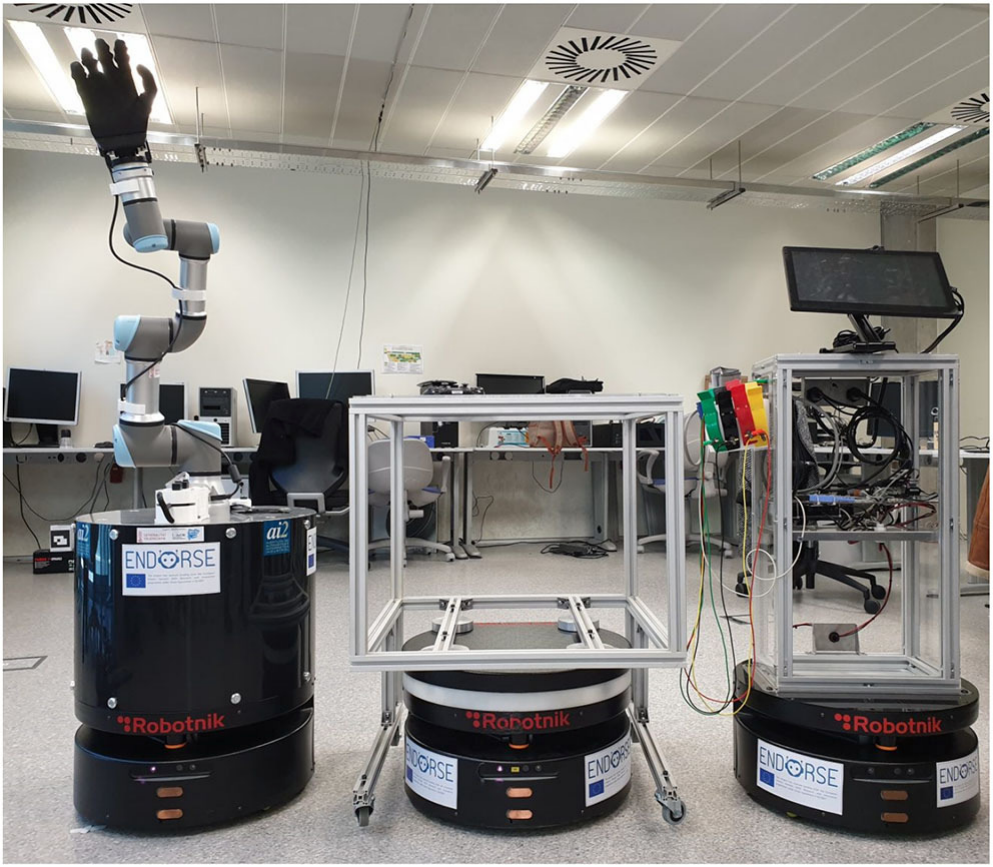
ENDORSE project proof-of-concept mobile robotic fleet [http://www.endorse-project.eu/]. Robotnik's RB-1 robot is used with different, easily swappable, and mountable hardware modules. From left to right, a robotic arm (Universitat Politecnica de Valencia, Spain), a carrier component for logistic tasks (Robotnik, Spain), and an e-diagnostic module for vital signs acquisition (StreamVision, France) and communication to a cloud-based electronic health record (EHR) system (University of Cyprus, Cyprus).

ENDORSE Concept is a European funded project aiming to address afore-described technological challenges and broaden the functional scope of mobile robotic solutions in indoor healthcare settings (36) (Figure 1). More specifically, innovation in ENDORSE is centered around the following four pillars:

- infrastructure-less indoor navigation of a mobile robots fleet;
- intelligent Human-Robot Interaction (HRI) toward optimizing the seamless sharing of crowded spaces between humans and robots;
- integration of ENDORSE software modules with corporate software solutions, complying with the latest EU regulations on data security;
- development of modular hardware mechanisms to accommodate a diverse set of tasks and services by simply swapping reconfigurable component modules.

ENDORSE functionality will be demonstrated via the integration of an e-diagnostic support module for vital signs monitoring on a fleet of mobile robots, facilitating connectivity to cloud-based Electronic Health Records (EHR), and validated in an operational hospital environment for realistic assessment.

### Survey on Nursing and Assistive Robots

Within the context of the ENDORSE project, a questionnaire was drafted aiming to capture end-users views upon different aspects of the ENDORSE concept in particular and robotics solutions in general. As such, the questionnaire was tailored with a focus primarily on nurses and secondarily on healthcare professionals and stakeholders, involved in the provision of clinical care in healthcare indoor settings. The latter, was the result of three focus groups that took place prior to finalizing the questionnaire. The first two focus groups involved senior nurses and experiences researchers, respectively, while the final one brought together both groups. The questionnaire was primarily circulated amongst the students and alumni of the Department of Nursing, Faculty of Health Sciences, of the Cyprus University of Technology (CUT) during September 2019. Secondarily, the questionnaire study involved experienced researchers in the broader electronic health and robotics areas. Here, it is important to highlight that a second questionnaire is scheduled as a part of ENDORSE research activities, aiming to capture the perceptions of patients. A total of 115 responders participated in the survey, of which more than 80% were nurses (16% of which were university students), 15% researchers with academic experience, 75% were aged between 18 and 34 years old, 75% were University graduates, and approximately two-thirds were females.

The questionnaire consisted of the following sections: (i) Demographics, (ii) Perceived behavioral control, (iii) Subjective norm, (iv) Safety and privacy considerations, (v) Operational perspective, and (vi) Management and financial perspective. An explanatory section highlighting the ENDORSE project's objectives (see section The ENDORSE Concept) preceded the survey questions, aiming to introduce the involved concepts to all participants. A link to the project's website and contact details for additional information were further provided. In what follows, key observations extracted from the analysis of 115 responses that were collected are described.

#### A Perceived Behavioral Control

**Figure 2:**
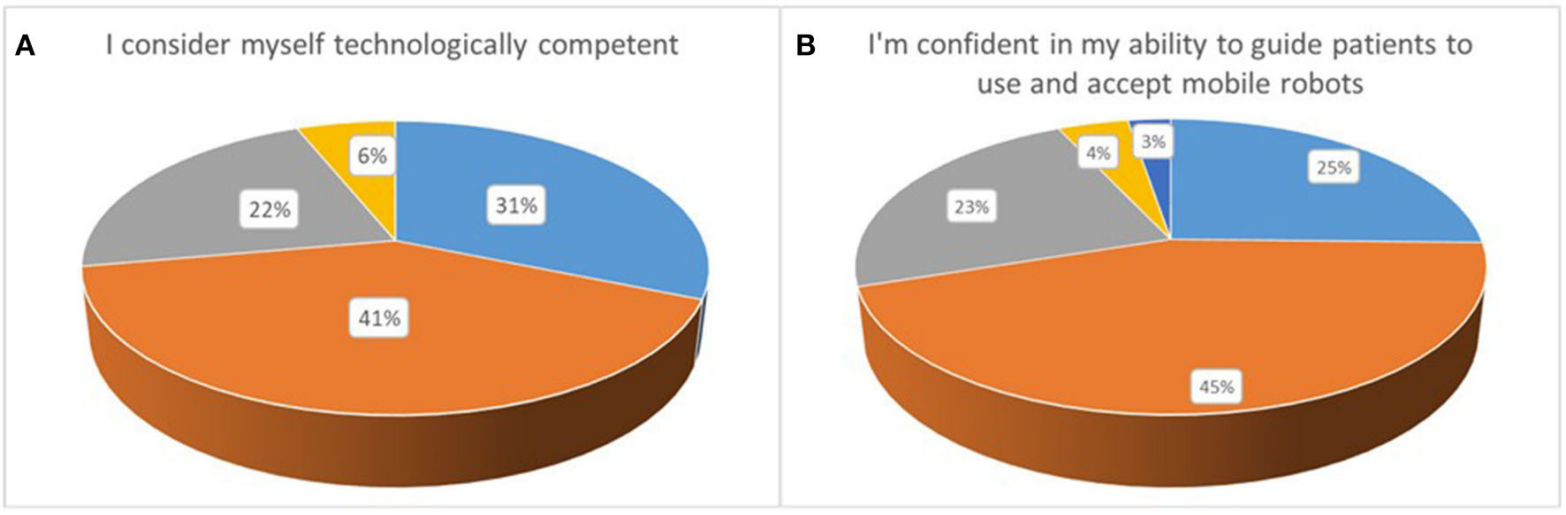
**(A,B)** Perceived behavioral control.

The opening section of the questionnaire consisted of 6 questions and its primary goal was to capture the end-users perceived behavioral control. A promising 72% of the participants, as depicted in Figure 2A, considered themselves technologically competent, while only 7 out of 115 participants (disagree: ~6%) considered themselves the opposite. Approximately 1/5 neither agreed nor disagreed with being technologically competent and familiar with technology. A very large percentage (~65%), received some form of robotic education/interaction during his/her undergraduate and graduate studies. This percentage aligns with the percentage of responders that stated being technologically competent. The latter emphasizes and reiterates the importance of bringing robotic education in university courses, and especially in health sciences and not just in computer science and engineering disciplines. Importantly, an impressive ~61% were aware of the DaVinci surgical robot, also showing the wide acceptance and penetration in clinical care this robotic solution enjoys over the past decade. Moreover, an interesting 36.5%, ~34, and 27%, were familiar with exoskeleton robotic solutions, robots used for assisted living applications, and mobile robots used for logistic applications, respectively. On the other hand, approximately a fifth of the participants were not aware of any of the listed robotic solutions. More than 80% expressed their strong confidence in their ability to learn how to interact and operate a mobile robot if one was to become a part of their healthcare unit. A similar percentage of ~73% strongly agreed or agreed that they are confident in their ability to learn how to guide their colleagues in operating mobile robots working in indoor healthcare spaces. Only 8.6% disagreed while 18.3% neither agreed nor disagreed. An elevated 23% (compared to the previous two questions) neither agreed nor disagreed with respect to the statement that they feel confident in guiding patients on how to use and accept the use of mobile robots in their daily care routine. On the opposite end, ~70% (strongly agree: ~25%; agree: ~45%) felt that learning how to guide patients coexist with indoor mobile robots should not be a challenge, as shown in Figure 2B. The last three questions do show a trend, that between 20 and 30% of responders are somehow skeptical with the idea of adopting a mobile robot in standard clinical care, with the underlying cause being how to convince patients or educate new colleagues in operating/interacting with mobile robots.

#### Subjective Norm

The subjective norm section of the questionnaire (7 questions) complements the perceived perspective, and was designed with the dual objective of first, capturing how healthcare professionals believe their colleagues would react in the adoption of mobile robots operating in a healthcare environment, and second, what they actually expect of that robot in practice.

**Figure 3:**
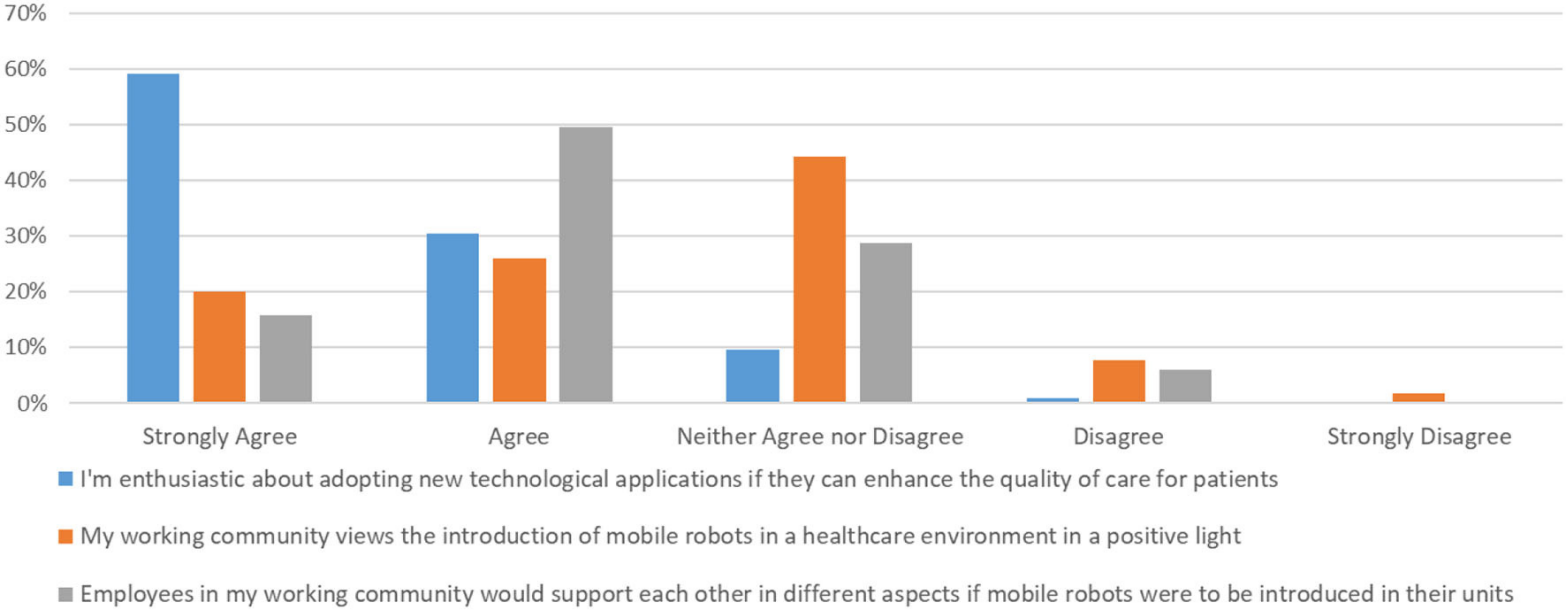
Subjective norm.

**Figure 4:**
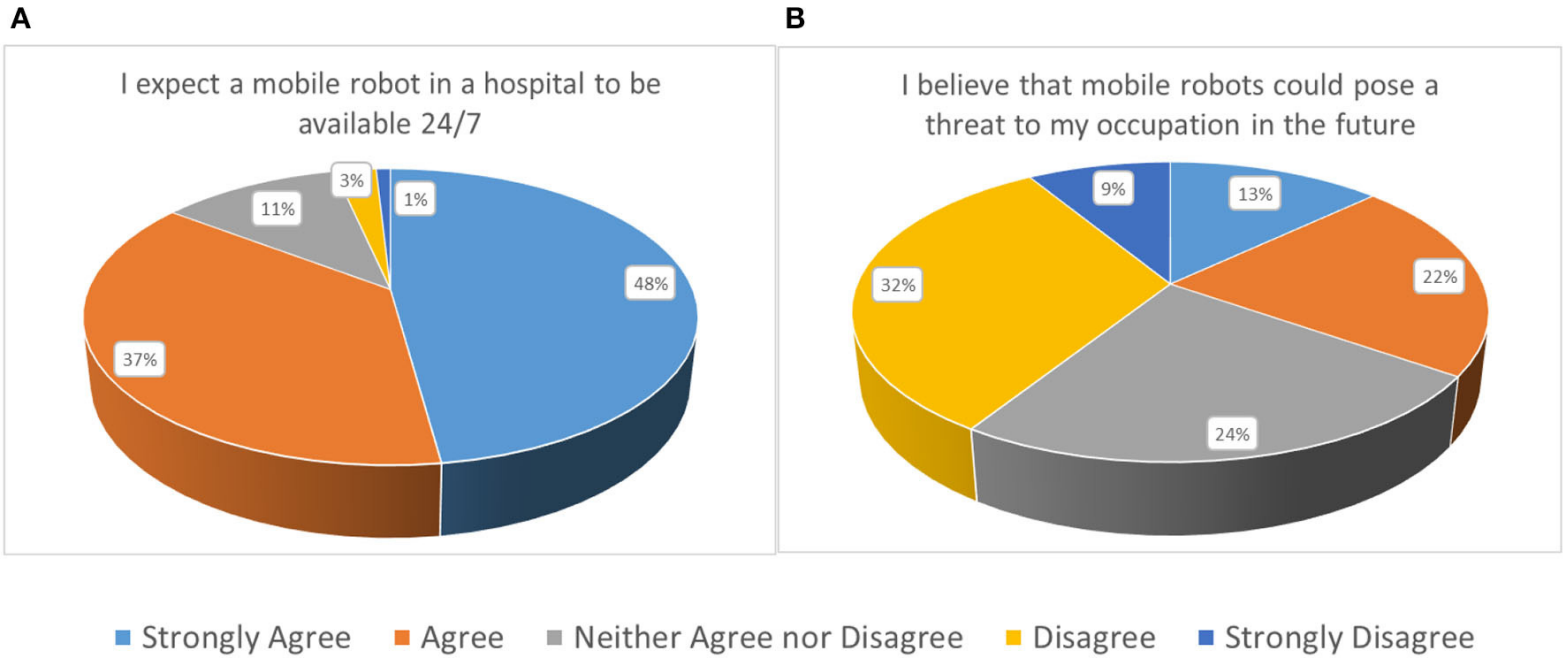
**(A)** Subjective norm and **(B)** Safety and privacy considerations.

An impressive 89.5% of the participants was enthusiastic of adopting an ENDORSE-like solution in their workplace, should this was linked to increasing the quality of the provided care, as highlighted in Figure 3. Importantly, there was only one response disagreeing on this particular question. However, in the following question, the participants appear to hesitate on how this would be received by their colleagues, with ~44% responding as neither agreeing nor disagreeing in the statement that their broader workplace sees the adoption of robotic solutions in indoor healthcare environments in a positive angle. Approximately 10% were pessimistic (believe the opposite) while ~46% were indeed optimistic (agree with the statement). The same trend was further documented in a follow-up question, trying to capture one's view with respect to their immediate colleagues. Again, 53% (against 44% in the previous question) selected a neutral response, depicting that they do believe that adopting mobile robots into daily healthcare routine and tasks is not a trivial task. More than one third however responded positively (strongly agree and agree: ~38%) while only a minor percentage of ~8% disagreed. In a similar question, that was phrased a bit differently, stating that colleagues would strongly resist the adoption of such solutions, a ~27% agreed (strongly agree: ~9%; agree: ~18%). This was the highest documented response for a potentially negative statement, showing that healthcare professionals do not take for granted that their colleagues share the same perceptions with respect to adopting a potentially transforming robotic solution. Still, the highest percentage of ~41% was neutral while almost 3 out of 10 (~29%) disagreed; being convinced that no technology-oriented opposition would appear. A great sign of solidarity was document in the next question, where about two thirds or ~68% responded that healthcare professionals would help each other, with any matter that should arise directly or indirectly, with the adoption of robotic solutions (see Figure 3). The next two questions revealed two key characteristics expected of robotic solutions that should be taken into careful consideration during design and development to facilitate user-acceptance. More specifically, ~76 and 85%, expect from a mobile robot to respond promptly to its tasks and be available 24/7, respectively (see Figure 4A). Only ~7 and 3.5% of responders did not have these expectations (the remaining being neutral), respectively.

#### Safety and Privacy Considerations

Six questions, addressing broader security (physical and cyber-security) and privacy considerations composed the present section of the questionnaire. The introductory question aimed at assessing whether the end-users felt that the introduction of mobile robots tasked with various operations that have so far been undertaken by clinical personnel (e.g., nurses), can potentially pose a threat to their occupation in the future. The responses were balanced, with 35% acknowledging that the latter scenario could become a reality, while 41% did not perceive the abovementioned statement as a threat, as demonstrated in Figure 4B. Approximately 24% gave a neutral response. Here, it is important to highlight an observed trend that has been evident throughout the questionnaire. Individuals that strongly feel technologically competent have a more positive predisposition and are typically less concerned with any negative developments that might arise from robotic solutions in a healthcare indoor setting. The opposite holds for individuals that do not feel adequately secured from a technological competencies angle.

**Figure 5:**
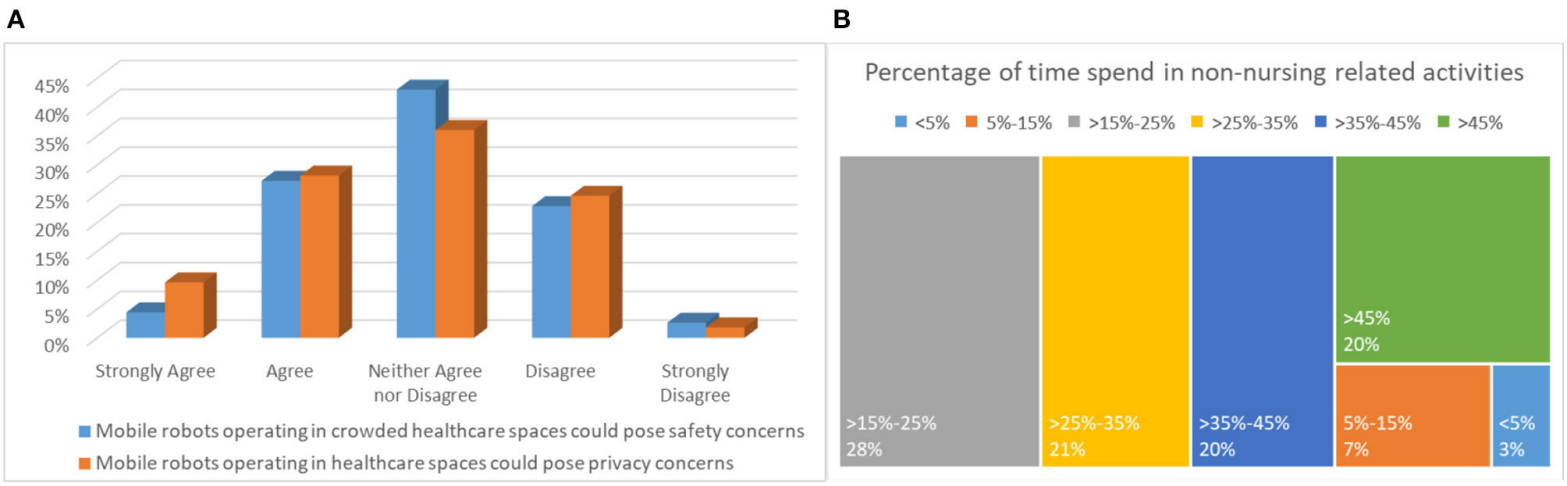
**(A)** Safety and privacy considerations and **(B)** Operational perspective.

The responses to the next question, whether such a development could incur any security issues, deliberately phrased in a high-level manner, were again balanced. The highest percentage, or 43%, gave neutral responses, while ~27 and 23% responded positively and negatively, respectively (see Figure 5A). Safer conclusions can be drawn from the next question, where specific examples concerning physical, infrastructure, and robot security were listed. More than half of the responders or ~56% considered the specific examples possible, which emphasizes that a secure-by-design robotic fleet development is of primary essence and a catalytic factor in user acceptance in a full-scale deployment scenario.

With respect to privacy considerations, responses to the general question if such a deployment could pose privacy issues, positive responses were slightly elevated compared to the corresponding question on security, as depicted in Figure 5A. In particular, ~38% believe that a privacy compromise is likely to occur, with 36% giving a neutral response, and ~27% (the same as above) giving a negative response (i.e., do not believe that a privacy breach is a true risk). Indeed, responses in the specific examples in the following question were slightly elevated. In fact, a ~37% was concerned of a privacy compromise of his/her personal data. Again, more than 4 out of 10 participants or ~43% considered that all listed privacy concerns are possible.

#### Operational Perspective

The operational perspective section consisted of 6 questions and provided significant insights with respect to the projected operational benefits of adopting an ENDORSE-like solution in routine daily care. An impressing 83.5% expects that physical burden will be significantly reduced, while 2/3 project that they will save time from tasks that do not relate to their primary mission of providing care, such as transfer of linens, food, waste, and other. In fact, only one responded that mobile robots are not suitable for such operations, demonstrating the physical and time burden experienced by healthcare professionals attributed to non-clinical tasks. Perhaps one of the most alerting responses of this questionnaire is associated to the next question, highlighted in Figure 5B, documenting the amount of time spent on non-clinical tasks. An extraordinary 89% responded that non-clinical tasks consume more than 15% of their time, of which ~65% more than 25%, ~40% more than 35%, and 20% more than 45%. The latter, is a key driving and motivating factor for designing efficient and effective robots that would assist healthcare professionals in their clinical, but more importantly, in their non-clinical tasks, allowing more time to be allocated for providing the appropriate levels of clinical care. Importantly, ~73% believe that patients would welcome mobile robots undertaking the above-mentioned non-clinical tasks.

**Figure 6:**
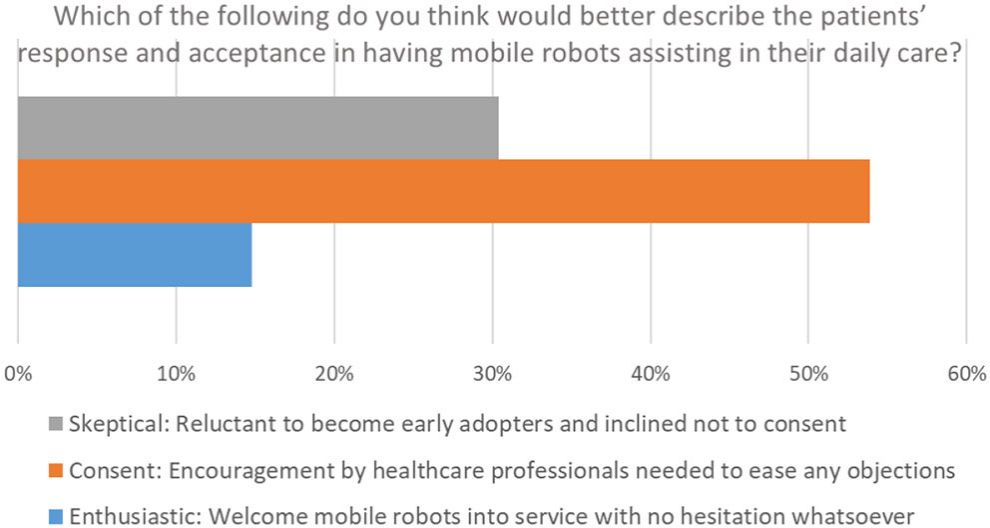
Operational perspective.

Further extending their acceptance to clinical tasks, more than half of the end-users participating in this survey, responded positively to the 5 listed clinical operations suggested in the next question. In fact, in 3 out of the 5 examples, the acceptance rate climbed to two out of three participants. In line with the aforementioned, 60% responded that they would trust a mobile robot undertaking certain clinical tasks such as the ones mentioned in this questionnaire (i.e., vital signs and medical data capture, electronic health records connectivity and medical data display, broader telemedicine and telehealth services, etc.), with only 10.5% declaring the opposite. Moreover, a noteworthy ~69% believe that patients would welcome and accept a robotic solution undertaking certain clinical tasks, should the involved healthcare professionals allocated the time to explain and convince them that such a development is for their own benefit (see Figure 6). On the other hand, ~31% replied that their feeling is that patients would be skeptical about such a scenario.

#### Management and Financial Perspective

The key objective of these two sections is to document an initial reaction of potential end-users concerning ENDORSE solution incurred costs as well as associated healthcare expenditures savings. While the questionnaire does not provide adequate data for a fair expenditure estimation, the documented opinions are nevertheless important for future reference. Moreover, a second objective was to document potential barriers to adoption. The first question indeed reveals the two most important barriers to wide mobile robots deployment in indoor healthcare spaces according to end-users. The first is the associated financial burden, with more than 80%, showing that based on the provided information, end-users expect ENDORSE solution to be costly. The second, with ~60%, is the patient acceptance, another key factor that has to be taken into consideration, provided that it is not technology-related. Other barriers that were ranked relatively high included end-user acceptance (~53%) and privacy issues (~52%).

Importantly, ~89% of the participants, were not aware of robotic solution like the one ENDORSE is proposing, highlighting the innovation potential of the proposed solution. However, for the financial part of this questionnaire, we opted to include only those who responded positively to the above question, in an attempt to receive more realistic cost estimations. Unfortunately, we only received 13 responses, of which ~46% anticipate the ENDORSE solution to cost between 100 and 200 k and another 23% more than 200 k. The latter suggests that robots are still considered an expensive technology and not a commodity. In the last question, results were balanced, with half of the responders believing that ENDORSE solution would save <100 k annually, for a 3-story hospital with 100 beds, and the other half more than a 100 k.

## The Challenges Ahead

Among the key challenges to successful implementations of nursing and assistive robotics is the user acceptance. Of relevance are the perceptions of patients/elderly as well as nurses/caregivers. The role of geographical and cultural differences may not be underestimated, as for example the case of China that is discussed in (37). On behalf of the patients/elderly there exists a natural concern that robot deployments at home may replace personal contact and assistance, leading to a loss of companionship and increased isolation. Robots also have the ability to learn and process personal information, which is in effect a privacy violation. The presence of robotic systems at home creates the feeling of being under continuous surveillance. Interestingly, from a different perspective, robotic technologies may in fact increase the level of privacy by avoiding the need for human assistance for tasks which are perceived as private. From a psychological perspective, a prominent non-physical risk is the attachment to the robot and deception about its abilities.

Many nursing professionals are accustomed to emerging technologies impacting their work and daily duties. Relevant robotics content is slowly becoming part of nursing education (38). However, an innate concern that the introduction of robotics is likely to threaten their job security may become an obstacle to the adoption of nursing robotic solutions. Along user acceptance, technological challenges pertinent to the robotics technology itself remain to be addressed. For mobile robots operating within crowded, dynamic spaces (e.g., a hospital or a house setup) (36), standard sensing, localization and navigation techniques as applied in structured environments (e.g., a production facility) are not readily applicable. Likewise, safety concepts applied to manipulation systems, which have been well-established in industrial setups, also need to be reconsidered. The ENDORSE concept has been contributing toward that direction. Specifications for nursing robots should include sterilizability so that the robot is prevented from becoming a contamination agent itself; this is particularly important in robot deployments during contagious disease outbreaks. Pertinent to communications and integration of cloud technologies, a potential safety risk related to robotic nursing is the possibility of unauthorized access to healthcare databases and sensitive private information; hence, data security technologies become relevant.

The success of nursing and elderly-care robots requires the safe and effective interaction between human and machine, which is a topic for human factors engineering. For elderly-care purposes, interfaces for human-robot interaction should be usable and appealing to older generations, also considering their relatively limited exposure to modern ICT applications. In that respect, personalization can be used, which is an advantage inherent in programmable robots. For an effective human factors design, a requirement is the sound understanding of the user characteristics, possibly associated to diseases, accidents, aging, and birth defects. Specific to elderly populations there exist three principal categories of disabilities (39): (1) Physical impairments, including motor limitations, limit an individual's ability to reach and manipulate controls. (2) Perceptual impairments (sensory limitations) impair an individual's ability to receive information and feedback. (3) Cognitive limitations impair an individual's ability to process information.

The appearance and aesthetics of physically and socially-assistive robots is in general considered important to users (23). Appearances may take different forms including machine-like, humanoid, and software agents with human faces. In the bibliography it has been widely recognized that a robot's physical appearance leads to social expectations; a human appearance may lead to unrealistic expectations beyond the actual capabilities of the robot (40). Regarding motion systems, the majority of mobile robots are wheeled, given the advantage of less mechanical and control complexity. Despite their complexity, a main advantage of legged/anthropomorphic robots is their readiness to operate in environments and use tools originally designed for humans.

To fulfill their duties both nursing and assistive robots require some degree of autonomy, but is important that high-level control remains in the hands of the user. Widening the use of autonomous robotic technologies will require a legal as well as ethical framework to provide a foundation for further developments. Among the unresolved issues is the attribution of civil and criminal liability should an autonomous robot produce damages (41, 42). The technological nature of nursing and care robots makes this issue rather complex.

Throughout the present study several key challenges pertinent to the introduction and use of nursing and assistive robotics have been identified and discussed, which eventually translate into corresponding design requirements. Informed design will constitute new robotic solutions more usable and effective, while facilitating acceptance by end-users. Toward that direction, the “design for X” (design for excellence) concept becomes relevant and it allows here to effectively summarize key requirements; the X variable is associated to different attributes of the system (e.g., safety) (43, 44). The identified design parameters are collected in Table 1. This design framework spans the whole life-cycle of nursing and assistive robots (the study outcomes provide input pertinent to the development and use phases). Noticeably, the requirements relevant to the use phase outnumber the requirements associated to the other life-cycle phases, which are mostly engineering and technological in nature. It is also pointed out that the compiled list of use phase requirements relates to the perspective of all stakeholders (nurses, patients, management).

**Table 1:** Design requirements summary using the “Design for X” framework.

**Life-cycle phase**	**X design parameter**		
Development	• Simplicity • Safety • Reliability • Quality	• Modularity • Reprogrammability • Interchangeability • Expandability	• Upgradability • Integrability • Standards/Regulations • Price
Production/Manufacturing	• Manufacturability • Assembly	• Testing • Integration	• Cost • Materials
Use	• Usability • Human Factors • Ergonomics • Error-Resistance • Aesthetics • User-Friendliness • Customizability • Personalization • Clinical Relevance	• Multi-Use • Autonomy • Energy Autonomy • Mobility and Speed • Maneuverability • Manipulability • Stability • Energy-Efficiency	• Cost Effectiveness • Load Capacity • Sterilizability • Maintainability • Serviceability • Physical Safety • Logistics • Cyber-Security • User Privacy • Ethics
Disposal	• Recyclability	• Reusability	• Sustainability

*The relevant X design parameters are grouped according to the life-cycle phase relevance*.

## Conclusions

Robots are currently impacting many aspects of our lives and their applications extend beyond their traditional applications in production. Nursing and assistive robotics are categorized within the broader scope of service robotics—the non-industrial uses of robots. In that context, autonomous and/or tele-operated robots, when employed in healthcare, can improve efficiency without compromising quality of care while reducing expenditures. Their mission further extends to elderly-care supporting the aging-in-place concept. Recently, an upgraded role for nursing robotics has emerged as effective means to combat outbreaks of infectious diseases. From a technical standpoint the involved technologies are mature. Yielding productive solutions necessitates integration of robotic components (mobile robots, manipulation systems, end-effectors, etc.) together with other enabling technologies (vision and image processing, video streaming, security, etc.). Toward this direction, robotic systems are inherently compatible and can be integrated with other contemporary technologies (i.e., internet-of-things, electronic health, etc.) to effectively increase their capabilities and clinical practice adoption.

Despite the potential of nursing and assistive robots there exist challenges that remain to be addressed prior to effective robot deployments of scale. Among key technological challenges, one can identify robot autonomy, indoor navigation and safe operation in healthcare settings. These areas are subject to further fundamental and applied scientific research. Inevitably, beyond the technological challenges, the perceptions and concerns of end-users toward these technologies will play a decisive role in future developments. The latter was the topic of the questionnaire study presented herein consolidating the nursing professionals' perspective on such pressing aspects as summarized next.

Interestingly, the majority of participants consider themselves technologically competent, confident with the idea of operating robots and interacting with them, as well as learning how to provide the required guidance to their colleagues and patients. Hence, it is no surprise that the same pool of responders appeared enthusiastic about adopting robotic solutions in their workplace. The latter, is rooted in the established expectations that robots will possess the ability to operate continuously throughout the day while promptly responding to the assigned tasks. In particular, nursing professionals anticipate that the adoption of robots in healthcare spaces will eventually alleviate the physical burden they currently experience that is attributed to non-clinical tasks, allowing them to concentrate on their primary clinical duties. Results highlighted that a considerable amount of time is actually consumed on often tedious, non-clinical tasks, such as logistics, transfers of linens, food and waste. In that context, the prevailing feeling among nursing professionals was that patients themselves would also be supportive of such robot implementations. Favorable responses were further recorded with respect to the acceptance of robots in reliably performing clinical tasks (e.g., capture of vital signs). The majority or responders believe that patients would react positively to the idea of robots undertaking clinical tasks, as long as healthcare professionals appropriately introduce the process.

In the opposite end, skepticism was indeed expressed by a certain percentage regarding the adoption of robots in clinical care due to the difficulties in convincing patients and educating new colleagues. The latter can be associated with the fact that responders did not have a clear view of whether their colleagues would indeed support such a transformative change. A significant concern that cannot be overlooked, although not being the prevalent impression, involves the scenario where robotic solutions pose a job security threat in the future. Toward this direction, primary user-acceptance concerns extend over security and privacy. Particular concerns have emerged with respect to both physical safety and cyber security. It is vital that these issues are thoroughly addressed via a secure-by-design approach (45–49). Likewise, concerns surfaced regarding the potential privacy compromises emanating from the presence of the robots and the corresponding sensitive data processing. To overcome these justified apprehensions, a clear and unambiguous regulatory framework overseeing nursing robot operations should be jointly developed by all involved stakeholders in collaboration with national authorities.

A key aspect affecting wider adoption in clinical practice involves the management and financial perspective. Demand exists for safe, reliable and cost-effective solutions, facilitating fast deployment and integration to existing IT infrastructure. However, the present robotic landscape market does not yet meet these expectations. As a result, there is a need for technological breakthroughs such as the ones pursued by the ENDORSE project that will remove existing financial barriers toward the large-scale adoption of nursing robots in healthcare environments. The realization of such advancements will in turn trigger the documented clinical and non-clinical benefits and effectively materialize reduction of healthcare expenditures in the near future.

The current review shows the potential that exists for nursing and assistive robotics, which was documented through the survey results. Clearly, challenges to be addressed extend beyond the technological and clinical issues to user acceptance. Despite an overall positive attitude that was recorded toward the introduction of robotics technologies some skepticism was also evident. Adequately addressing these concerns will be important for their future and informed robot design becomes critical toward that direction.

To promote the use of these technologies there exist three axes for targeted action, directed toward the end-users. Firstly, nursing and assistive robotics can become part of the nursing professionals' education, familiarizing and allowing them to effectively utilize these tools but also appropriately present them to patients and older adults. It is important that the capabilities of the robots are clarified and their role is transparent to the users. The second direction is to ensure direct involvement of all stakeholders in the product development stage, with patients' associations engaged in a leading role. Beyond the desirable effect on user acceptance it will eventually result to more efficient clinically-oriented solutions. Finally, wider adoption of nursing and assistive robotics will depend on successful implementations and demonstrations in clinical practice, while keeping in mind that evaluation will be on the basis of healthcare quality and cost effectiveness.

## Data Availability Statement

The raw data supporting the conclusions of this article will be made available by the authors, without undue reservation.

## Author Contributions

All authors listed have made a substantial, direct and intellectual contribution to the work, and approved it for publication.

## Conflict of Interest

The authors declare that the research was conducted in the absence of any commercial or financial relationships that could be construed as a potential conflict of interest.

## References

[1] Albertone G Allen S Redpath A. Ageing Europe. 2019 ed, Luxembourg: Publications Office of the European Union (2019).

[2] United Nations. World Population Ageing. United Nations, Department of Economic and Social Affairs (2019).

[3] Kumar ES Sachin P Vignesh BP Ahmed MR. Architecture for IOT based geriatric care fall detection and prevention. In: Proceedings of the 2017 International Conference on Intelligent Computing and Control Systems (Madurai: ICICCS). (2017). p. 1099–104.

[4] Christoforou EG Panayides AS Avgousti S Masouras P Pattichis CS. An overview of assistive robotics and technologies for elderly care. IFMBE Proc. (2020) 76:971–6. 10.1007/978-3-030-31635-8_118

[5] Uddin Z Khaksar W Torresen J. Ambient sensors for elderly care and independent living: a survey. Sensors. (2018) 18:2027. 10.3390/s18072027 29941804 PMC6068532

[6] Tsukiyama T. In-home health monitoring system for solitary elderly. Proc Comput Sci. (2015) 63:229–35. 10.1016/j.procs.2015.08.338

[7] Avgousti S Christoforou EG Panayides AS Masouras P Vieyres P Pattichis CS. Robotic systems in current clinical practice. In: 20th IEEE Mediterranean Eletrotechnical Conference IEEE MELECON (Palermo: IEEE) (2020).

[8] Turja T Taipale S Kaakinen M Oksanen A. Care workers' readiness for robotization: identifying psychological and socio-demographic determinants. Int J Soc Robot. (2020) 12:79–90. 10.1007/s12369-019-00544-9

[9] Tashiro T Aoki K Lee Y Sakaki T. Research and development of wearable auxiliary tool for behavior assistance of elderly who requires nursing care. In: International Conference on Control, Automation and Systems. Jeju: IEEE Computer Society (2017). p. 1501–4.

[10] Kyriacou E Pattichis MS Pattichis CS Panayides A Pitsillides A. M-health e-emergency systems: current status and future directions. IEEE Antennas and Propagation Magazine (2007, June 11).

[11] Tapus A Mataric MJ Scassellati B. Socially assistive robotics [grand challenges of robotics]. IEEE Robot Automat Mag. (2007) 14:35–42. 10.1109/MRA.2007.339605

[12] Portugal D Alvito P Christodoulou E Samaras G Dias J. A study on the deployment of a service robot in an elderly care center. Int J Soc Robot. (2019) 11:317–41. 10.1007/s12369-018-0492-5

[13] Marti P Bacigalupo M Giusti L Mennecozzi C Shibata T. Socially assistive robotics in the treatment of behavioural and psychological symptoms of dementia. In: Proceedings of the First IEEE/RAS-EMBS International Conference on Biomedical Robotics and Biomechatronics, 2006. BioRob 2006. Pisa: IEEE (2006). p. 483–8.

[14] Kramer SC Friedmann E Bernstein PL. Comparison of the effect of human interaction, animal-assisted therapy, and AIBO-assisted therapy on long-term care residents with dementia. Anthrozoös. (2009) 22:43–57. 10.2752/175303708X390464

[15] Shibata T. Therapeutic seal robot as biofeedback medical device: qualitative and quantitative evaluations of robot therapy in dementia care. Proc IEEE. (2012) 100:2527–38. 10.1109/JPROC.2012.2200559

[16] Begum M Wang R Huq R Mihailidis A. Performance of daily activities by older adults with dementia: the role of an assistive robot. IEEE Int Conf Rehabil Robot. (2013) 2013:6650405. 10.1109/ICORR.2013.6650405 24187224

[17] Fasola J Matarić MJ. Using socially assistive human-robot interaction to motivate physical exercise for older adults. Proc IEEE. (2012) 100:2512–26. 10.1109/JPROC.2012.2200539

[18] Görer B Salah AA Akin HL. A robotic fitness coach for the elderly. Lect Notes Comput Sci. (2013) 8309:124–39. 10.1007/978-3-319-03647-2_9

[19] Balaguer C Gimenez A Jardon A Cabas R Correal R. Live experimentation of the service robot applications for elderly people care in home environments. In: 2005 IEEE/RSJ International Conference on Intelligent Robots and Systems. Edmonton, AB: IEEE, IROS (2005). p. 2733–8.

[20] Takahara S Jeong S. Prototype design of robotic mobility aid to assist elderly's standing-sitting, walking, and wheelchair driving in daily life. In: International Conference on Control, Automation and Systems. Seoul: IEEE Computer Society (2014). p. 470–3.

[21] Carlson T Demiris Y. Collaborative control for a robotic wheelchair: evaluation of performance, attention, and workload. IEEE Trans Syst Man Cybern B Cybern. (2012) 42:876–88. 10.1109/TSMCB.2011.2181833 22275718

[22] Candiotti JL Daveler BJ Kamaraj DC Chung CS Cooper R Grindle GG. A heuristic approach to overcome architectural barriers using a robotic wheelchair. IEEE Trans Neural Syst Rehabil Eng. (2019) 27:1846–54. 10.1109/TNSRE.2019.2934387 31403434

[23] Hersh M. Overcoming barriers and increasing independence–service robots for elderly and disabled people. Int J Adv Robot Syst. (2015) 12:114. 10.5772/59230

[24] Ktistakis IP Bourbakis NG. A survey on robotic wheelchairs mounted with robotic arms. In: 2015 National Aerospace and Electronics Conference (NAECON). Dayton, OH: IEEE (2015). p. 258–62.

[25] Panayides AS Pattichis MS Pantziaris M Constantinides AG Pattichis CS. The battle of the video codecs in the healthcare domain - a comparative performance evaluation study leveraging VVC and AV1. IEEE Access. (2020) 8:11469–81. 10.1109/ACCESS.2020.2965325

[26] Avgousti S Christoforou EG Panayides AS Voskarides S Novales C Nouaille L. Medical telerobotic systems: current status and future trends. BioMed Eng. (2016) 15:96. 10.1186/s12938-016-0217-7 27520552 PMC4983067

[27] Zhou H Yang G Lv H Huang X Yang H Pang Z. IoT-enabled dual-arm motion capture and mapping for telerobotics in home care. IEEE J Biomed Health Inform. (2020) 24:1541–9. 10.1109/JBHI.2019.2953885 31751288

[28] Reis A Xavier R Barroso I Monteiro MJ Paredes H Barroso J. The usage of telepresence robots to support the elderly. In: 2018 2nd International Conference on Technology and Innovation in Sports, Health and Wellbeing (TISHW). Thessaloniki: IEEE (2018). p. 1–6.

[29] Li Z Moran P Dong Q Shaw RJ Hauser K. Development of a tele-nursing mobile manipulator for remote care-giving in quarantine areas. In: Proceedings - IEEE International Conference on Robotics and Automation. Singapore: Institute of Electrical and Electronics Engineers Inc. (2017). p. 3581–6.

[30] Yang GZ Nelson BJ Murphy RR Choset H Christensen H Collins SH. Combating COVID-19-the role of robotics in managing public health and infectious diseases. Sci Robot. (2020) 5:eabb5589. 10.1126/scirobotics.abb5589 33022599

[31] Vieyres P Poisson G Courreges F Merigeaux O Arbeille P. The TERESA project: from space research to ground tele-echography. Ind Robot. (2003) 30:77–82. 10.1108/01439910310457742 12807138

[32] Vieyres P Poisson G Courrèges F Smith-Guerin N Novales C Arbeille P. A tele-operated robotic system for mobile tele-echography: the Otelo project. M-Health. (Boston, MA: Springer) (2006) 461–73. 10.1007/0-387-26559-7_35

[33] Bonaci T Herron J Yusuf T Yan J Kohno T Chizeck HJ. To make a robot secure: an experimental analysis of cyber security threats against teleoperated surgical robots. arXiv[Preprint].arXiv:1504.04339. (2015).

[34] ENISA. Cyber Security and Resilience for Smart Hospitals (2016).

[35] Reportsanddata. Healthcare Cybersecurity Market to Reach USD 27.1 Billion by 2025. (2019). Available online at: https://www.globenewswire.com/news-release/2019/08/26/1906602/0/en/Healthcare-Cybersecurity-Market-To-Reach-USD-27-10-Billion-By-2026-Reports-And-Data.html

[36] Ramdani N Panayides A Karamousadakis M Mellado M Lopez R Christophorou C. A safe, efficient and integrated indoor robotic fleet for logistic applications in healthcare and commercial spaces: the endorse concept. In: Proceedings - IEEE International Conference on Mobile Data Management. Hong Kong: IEEE (2019). p. 425–30.

[37] Sifeng Z Min T Zehao Z Zhao Y. Capturing the opportunity in developing intelligent elderly care robots in china challenges, opportunities and development strategy. In: Proceedings of IEEE Workshop on Advanced Robotics and Its Social Impacts, ARSO. Shanghai: IEEE Computer Society (2016). p. 61–6.

[38] Mudd SS McIltrot KS Brown KM. Utilizing telepresence robots for multiple patient scenarios in an online nurse practitioner program. Nurs Educ Perspect. (2019) 41:260–62. 10.1097/01.NEP.0000000000000590 31714434

[39] Denno S Isle BA Ju G Koch CG Metz SV Penner R. Human Factors Design Guidelines for the Elderly and People with Disabilities. Technical Report SSDC-SYS/AI-C92-009. Minneapolis, MN: Honeywell (1992).

[40] Feil-Seifer D Mataric M. Socially assistive robotics. IEEE Robot Autom Mag. (2011) 18:24–31. 10.1109/MRA.2010.940150

[41] Brozek B Jakubiec M. On the legal responsibility of autonomous machines. Artif Intell Law. (2017) 25:293–304. 10.1007/s10506-017-9207-8

[42] Abbott R Borges G Dacoronia E Devillier N Jankowska-Augustyn M Karner E. Liability for Artificial Intelligence and Other Emerging Digital Technologies. Report from the Expert Group on Liability and New Technologies – New Technologies Formation European Union. (2019). Available online at: https://ec.europa.eu/transparency/regexpert/index.cfm?do=groupDetail.groupMeetingDoc&docid=36608

[43] Bralla JG. Design for Excellence. New York, NY: McGraw-Hill Professional Publishing (1996).

[44] Pahl G Beitz W. Engineering Design - A Systematic Approach. 2nd ed. London; Berlin; New York, NY: Springer Science & Business Media (1996).

[45] Dieber B Breiling B Taurer S Kacianka S Rass S Schartner P. Security for the robot operating system. Robot Auton Syst. (2017) 98:192–203. 10.1016/j.robot.2017.09.017

[46] Dieber B White R Taurer S Breiling B Caiazza G Christensen H. Penetration testing ROS. Stud Computat Intell. (2020) 831:183–225. 10.1007/978-3-030-20190-6_8

[47] Guerrero-Higueras ÁM DeCastro-García N Matellán V. Detection of cyber-attacks to indoor real time localization systems for autonomous robots. Robot Auton Syst. (2018) 99:75–83. 10.1016/j.robot.2017.10.006

[48] Vilches VM Gil-Uriarte E Ugarte IZ Mendia GO Pisón RI Kirschgens LA. Towards an open standard for assessing the severity of robot security vulnerabilities, the Robot Vulnerability Scoring System (RVSS). arXiv[Prerpint].arXiv:1807.10357. (2018).

[49] Vilches VM Kirschgens LA Calvo AB Cordero AH Pisón RI Vilches DM. Introducing the Robot Security Framework (RSF). p. a standardized methodology to perform security assessments in robotics. arXiv[Preprint].arXiv:1806.04042. (2018).

